# Optic Coherence Angiography Findings in Type-2 Macular Telangiectasia

**DOI:** 10.4274/tjo.68335

**Published:** 2017-10-27

**Authors:** Hilal Nalcı, Figen Şermet, Sibel Demirel, Emin Özmert

**Affiliations:** 1 Ankara University Faculty of Medicine, Department of Ophthalmology, Ankara, Turkey

**Keywords:** Macular telangiectasia type-2, optic coherence tomography angiography, spectral domain optic coherence tomography

## Abstract

**Objectives:**

To evaluate the vascular changes of idiopathic macular telangiectasia type 2 (MacTel 2) patients with optical coherence tomography angiography (OCTA) and correlate these changes with the findings of spectral domain optical coherence tomography (SD-OCT).

**Materials and Methods:**

Simultaneous SD-OCT and OCTA images of 10 eyes of 6 patients who were diagnosed as MacTel 2 in Ankara University Faculty of Medicine, Department of Ophthalmology were obtained and graded according to the OCTA grading system for MacTel 2.

**Results:**

Ten eyes of 6 patients were included. Four (66%) patients were female and 2 (34%) were male. The disease was grade 0 in 2 eyes, grade 1 in 2 eyes, grade 2 in 3 eyes, grade 3 in 1 eye, grade 4 in 1 eye, and grade 5 in 1 eye. The most common findings in grade 1, 2, and 3 non-proliferative disease were thinning of the outer retinal layers, presence of intraretinal hyporeflective layers and inner limiting membrane draping. In cases with subretinal choroidal neovascularisation (CNV) in OCTA, CNV or CNV scar was present in the B-scan SD-OCT images. In a case in which OCT was within normal limits, vascular changes consistent with grade 1 disease were observed in OCTA. On the contrary, 2 patients with significant foveal atrophy and macular hole in B-scan showed changes of early disease in OCTA. In some of the eyes, OCTA revealed an intact superficial vascular layer despite visible changes in the deep layer and the presence of neovascularisation.

**Conclusion:**

OCTA yields findings which are important for understanding the pathogenesis of the disease and providing better follow-up. Contrary to fundus fluorescein angiography, changes in the deep arterial plexus in the early disease and CNV can be clearly observed with OCTA. To achieve the best results in clinical practice, en face flow maps should be evaluated together with B-scan SD-OCT images.

## INTRODUCTION

Idiopathic macular telangiectasia type 2 (MacTel 2 or perifoveal telangiectasia) is an acquired vascular disease of the macula primarily involving neural and glial cell degeneration and loss. This condition, named by Yannuzzi et al.,^1^ refers to idiopathic juxtaretinal telangiectasia (IJRT) type 2A according to the classification system introduced by Gass and Blodi^2^ in 1993. It is the most common type of IJRT, and includes cases that are usually bilateral, occult, and nonexudative. The disease typically presents in the fifth and sixth decades and affects men and women equally, although various studies have reported contradictory findings on the latter point.^3,4,5^

Clinically, it is characterized by loss of transparency in the foveal region, intraretinal crystalline deposit accumulation, hyperplastic retinal pigment epithelium migration, macular pigment loss, and progressive abnormalities in the juxtafoveolar retinal vessels. These include right-angle veins, subretinal and outer retinal neovascularization, and vascular invasion of the foveal avascular zone.^6^ Optical coherence tomography (OCT) allows better understanding of retinal pathologies. Characteristic findings of the disease include macular thinning, hyporeflective cavitations in the inner and outer retinal layers, development of full-thickness macular hole in the absence of vitreoretinal traction, and atrophic changes in the outer retinal layer.^7,8^

From its initial description to the present day, there has been no consensus regarding the mechanism of perifoveal telangiectasia. However, it is currently believed that the degeneration of Müller cells, which serve protective and supportive functions, is the primary pathology, and the damage to the retinal layers, atrophy, and vascular changes develop secondarily.^3^

Optical coherence tomography angiography (OCTA) is an imaging method that visualizes the movements of blood cells using motion contrast, thus providing information about blood flow. Thusly, imaging of the retinal vascular layers is possible without the need for intravenous contrast injection. As opposed to fundus fluorescein angiography (FFA), in which layers other than the superficial vascular plexus appear only as background hyperfluorescence, OCTA enables separate imaging of the superficial and deep vascular layers and the choroidal vessels.^9^

In this study, we aimed to use OCTA to evaluate vascular anomalies in patients with MacTel 2 and elucidate the relationship between these and retinal anomalies detected on OCT.

## MATERIALS AND METHODS

Optovue spectral domain OCT (SD-OCT) and OCTA images were recorded simultaneously from 6 patients diagnosed with MacTel 2 in the Ophthalmology Department of the Ankara University Faculty of Medicine. Patients were considered to have MacTel 2 in the presence of findings such as reduced autofluorescence in the central macula on fundus autofluorescence; loss of retinal transparency, intraretinal pigment, presence of right-angle venules, and telangiectatic vessel appearance on fundus examination; and OCT showing interior limiting membrane drape, retinal pigment epithelium migration creating intraretinal hyperreflectivity, atrophy of the outer retinal layers, and presence of intraretinal hyporeflective cavitation. OCTA en face flow maps were created using RTVue XR Avanti version 2015.1.1.98 and Split Spectrum Amplitude Decorrelation Angiography images were obtained. Images that could not be evaluated due to low resolution were excluded. Each eye was graded based on the MacTel OCTA grading system according to Chen et al.^10^ (Table 1).

## RESULTS

**Figure 1:**
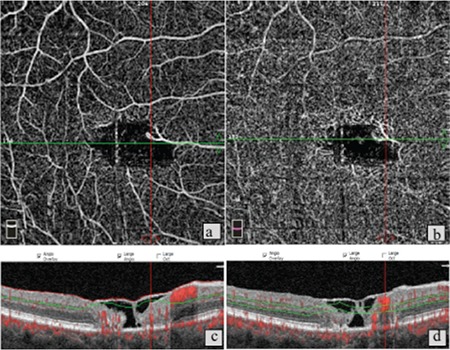
Patient 6, right eye. a) Foveal avascular zone (FAZ) irregularity and reduced vascular density in the superficial vascular network; b) Optical coherence tomography angiography in the same eye shows stage 3 disease with irregularity and vascular invasion of the FAZ; c,d) B-scan imaging shows foveal atrophy accompanied by outer retinal atrophy, inner segment/outer segment band defect, and stage 2 macular hole without internal limiting membrane drape or traction

**Figure 2:**
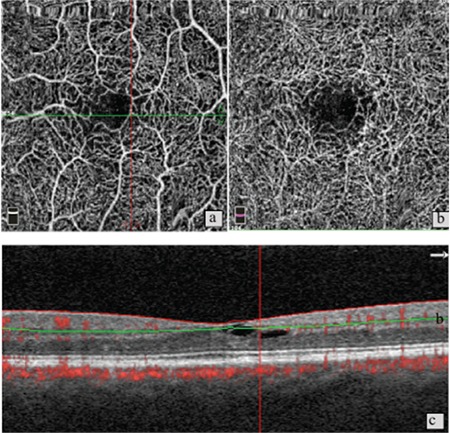
Patient 1, left eye, stage 2. a) Optical coherence tomography angiography shows minimal increase in space between vessels in the superficial vascular network; b) in the same eye, increased space between vessels and telangiectatic changes in the deep vascular network; c) B-scan imaging shows intraretinal hyperreflective cavitations

**Figure 3:**
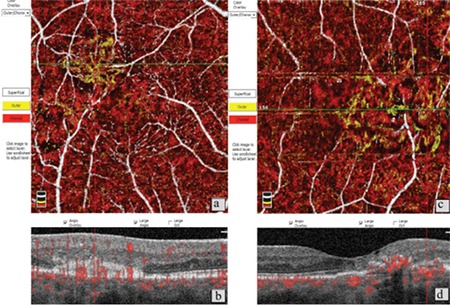
a) Patient 4, right eye, stage 4. Optical coherence tomography angiography shows neovascularization in the deep capillary network; b) B-scan optical coherence tomography in the same eye reveals classic choroidal neovascularization; c) Same patient’s left eye, stage 5. d) OCT shows pronounced atrophy of the outer retinal layers and a large, scarred fibrovascular membrane

**Figure 4:**
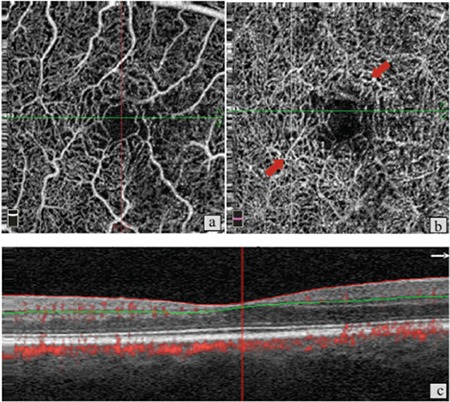
Patient 1, right eye. a) Superficial capillary plexus appears normal on optical coherence tomography angiography imaging; b) Widened vessels and telangiectases are apparent in the deep vascular network; c) B-scan optical coherence tomography imaging is within normal limits

**Figure 5:**
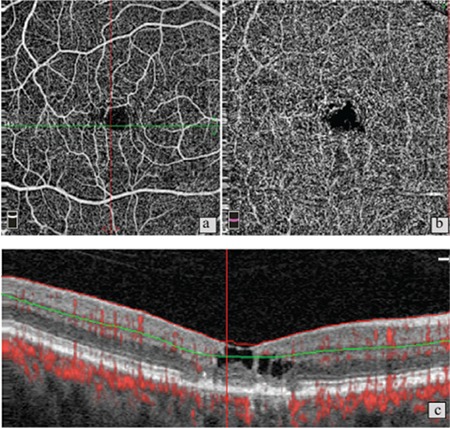
Patient 3, right eye. a) Capillary plexus appears within normal limits on optical coherence tomography angiography imaging; b) No findings other than minimal vessel thickening in the deep layer; c) B-scan OCT imaging shows pronounced internal limiting membrane drape, cavitation, and atrophy in the outer segments

**Figure 6:**
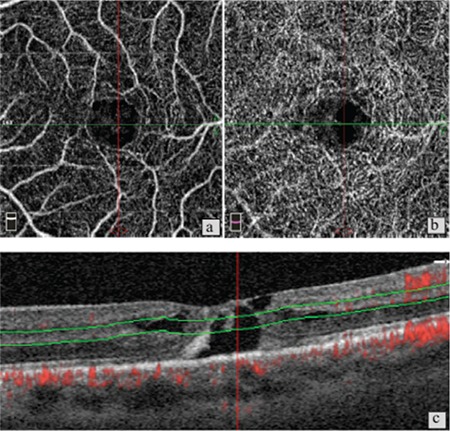
Patient 2, right eye, stage 1. a) Optical coherence tomography angiography reveals no pronounced changes in the superficial capillary network; b) Mild telangiectatic changes in the deep capillary plexus; c) B-scan imaging shows marked intraretinal cavitation and stage 1 macular hole

**Figure 7:**
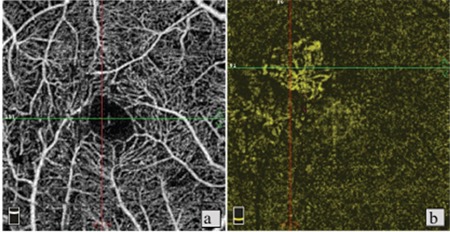
Patient 4, right eye. a) No pronounced changes in the superficial vascular layer; b) Neovascularization in the normally avascular outer retinal area

**Figure 8:**
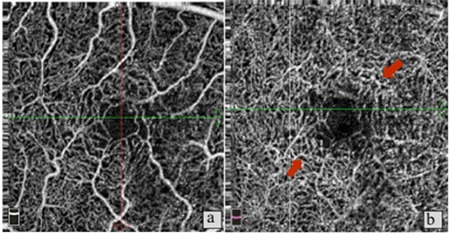
Patient 1, right eye. A) Superficial capillary layer appears normal; B) Thickening and telangiectases in the vessels of the deep capillary network

Ten eyes of 6 patients were included in the study. Four (66%) of the patients were female, 2 (34%) were male. Their mean age was 67.6 years. The OCT and OCTA findings of the patients are shown in Table 2. Disease severity was grade 0 in 2 eyes, grade 1 in 2 eyes, grade 2 in 3 eyes, grade 3 in 1 eye, grade 4 in 1 eye, and grade 5 in 1 eye. Neovascularization was not observed in any of the grade 1, 2, or 3 eyes. In these cases, the most common findings on OCT images were thinning and atrophy in the outer retina and inner/outer segment layers, intraretinal hyporeflective cavitations, and ILM drape (Figures 1 and 2). In patients with subretinal neovascularization apparent on OCTA, B-scan images from the same cross-section revealed classic CNV or CNV scar (Figure 3). In one patient, OCT images were considered normal, whereas vascular changes consistent with grade 1 disease were observed on OCTA (Figure 4). In contrast, 2 patients who showed pronounced foveal atrophy and macular hole on B-scan imaging had only early-stage changes in OCTA (Figures 5 and 6). Another striking finding was that in some of the patients, the superficial layer was intact despite pronounced changes and neovascularization in the deep layer (Figures 7 and 8).

## DISCUSSION

OCTA provides valuable information regarding disease severity and neovascularization activity in patients with parafoveal telangiectasia. In a study using OCTA, Spaide et al.^9^ demonstrated that FFA primarily shows the superficial vascular network of the retina and is inadequate for visualizing the deep capillary layer and the choroid. Consistent with other OCTA studies, in our series we observed that the superficial vascular network of some MacTel 2 patients was remarkably well preserved, even in advanced disease, while pronounced changes were evident in the deep capillary network. In addition, abnormalities may occur in the deep vascular network even in early disease stages where OCT is within normal limits, and FFA alone is not sufficient to evaluate these patients. It has also been shown that FFA findings which may be evaluated as perifoveal vascular leakage and lead to unnecessary injections in clinical practice are due to telangiectatic vessels in the deep vascular layer.^11^ OCTA clearly demonstrates that leakage simulating choroidal neovascularization on FFA is not a result of the formation of new vessels. Other advantages over FFA are that it is easy to perform, can be repeated frequently, and is non-invasive.

In the current series, OCTA evaluation of patients with advanced atrophic changes in the foveal region and macular hole revealed minimal changes consistent with grade 0 or 1 disease in the foveal vascular network. These findings support the theory that retinal structural changes seen in MacTel 2 are not secondary to vascular anomalies, but are a primary condition.^12,13^ Müller cells are known to play important roles in the maintenance of foveal structural integrity, neuronal support, and continuity of the blood-retina barrier.^14,15^ It is believed that macular hole and intraretinal cavitation arise due to neural atrophy and disruption of the foveal structure resulting from the destruction of Müller cells.^13^ These findings appear independent of vascular changes and show the clinical importance of en face circulation maps in combination with B-scan OCT.

On the other hand, a patient with grade 2 disease in the left eye exhibited no pronounced abnormality in the right eye on OCT, while OCTA revealed reduced vascular density and telangiectatic vessels in the deep vascular plexus. On careful examination of SD-OCT images of this eye, it was noted that the foveal pit was asymmetric. Similarly, Charbel Issa et al.^16^ studied patients with marked MacTel 2 in one eye and apparently healthy fellow eyes and detected foveal pit asymmetry and temporal foveal thinning which were associated with very early disease.

Another finding we observed in our cases is that the formation of new vessels was not limited to the choroid, but could also occur between the outer retinal layer and the choroidal vascular network, which is usually avascular. This finding is also similar to those observed by Spaide et al.^17,18^ In their OCTA studies of MacTel 2, they emphasized the antiangiogenic properties of Müller cells, stating that hypoxia due to deep vascular network dysfunction and suppression of antiangiogenic factors due to Müller cell degeneration may lead to intraretinal neovascularization. Several previous studies have indicated that neovascularization in MacTel 2 originates more from retinal vascular structures rather than the choroid.^19,20^ In their study using OCTA, Zhang et al.^21^ showed that these subretinal neovascular complexes may not be only retinal, but may also be connected to the choroidal vasculature.

The fact that the severity of intraretinal and vascular changes are mutually independent supports the view that the primary pathology of MacTel 2 is not vascular. Moreover, it has been shown that Müller cell loss may be another factor that leads to telangiectasia.^22^ Spaide et al.^17,18^ also emphasized the importance of Müller cells in intraretinal vascular circulation and retinal support. Thus, it seems that Müller cell loss is responsible not only for intraretinal neural cell death and morphologic changes, but also vascular telangiectasia, leakage, and neovascularization.

## CONCLUSION

The results of this pilot study demonstrate that OCTA, which has recently been introduced into clinical use, offers insight into the pathogenesis of MacTel 2 and is useful as an auxiliary method to OCT in patient follow-up, but does not directly correlate with OCT findings in terms of the extent of anatomic abnormalities.

## Figures and Tables

**Table 1:**
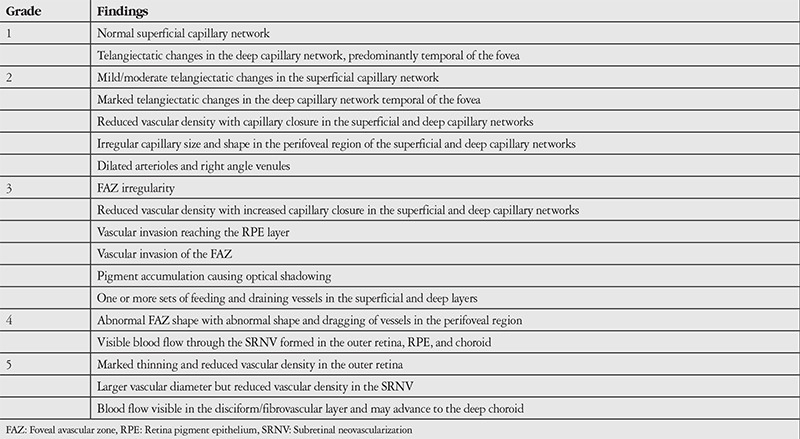
Staging of idiopathic macular telangiectasia type 2 patients based on optical coherence tomography angiography imaging

**Table 2:**
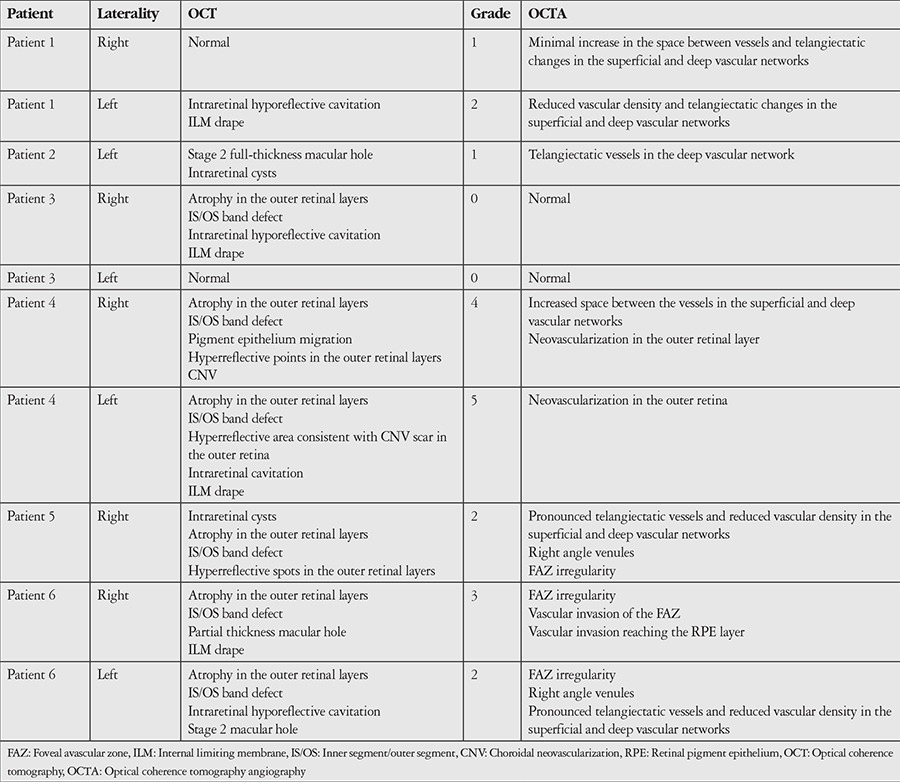
Spectral domain optical coherence tomography and optical coherence tomography angiography findings of the patients

## References

[1] Yannuzzi LA, Bardal AM, Freund KB, Chen KJ, Eandi CM, Blodi B (2006). Idiopathic macular telangiectasia. Arch Ophthalmol..

[2] Gass JD, Blodi BA (1993). Idiopathic juxtafoveolar retinal telangiectasis. Update of classiﬁcation and follow-up study. Ophthalmology..

[3] Wu L, Evans T, Arevalo JF (2013). Idiopathic macular telangiectasia type 2 (idiopathic juxtafoveolar retinal telangiectasis type 2A, Mac Tel 2). Surv Ophthalmol..

[4] Lee SW, Kim SM, Kim YT, Kang SW (2011). Clinical features of idiopathic juxtafoveal telangiectasis in Koreans. Korean J Ophthalmol..

[5] Aung KZ, Wickremasinghe SS, Makeyeva G, Robman L, Guymer RH (2010). The prevalence estimates of macular telangiectasia type 2: the Melbourne Collaborative Cohort Study. Retina..

[6] Gass JD, Oyakawa RT (1982). Idiopathic juxtafoveolar retinal telangiectasis. Arch Ophthalmol..

[7] Karth PA, Raja SC, Brown DM, Kim JE (2014). Outcomes of macular hole surgeries for macular telangiectasia type 2. Retina..

[8] Cohen SM, Cohen ML, El-Jabali F, Pautler SE (2007). Optical coherence tomography ﬁndings in nonproliferative group 2a idiopathic juxtafoveal retinal telangiectasis. Retina..

[9] Spaide RF, Klancnik JM, Cooney MJ (2015). Retinal vascular layers imaged by fluorescein angiography and optical coherence tomography angiography. JAMA Ophthalmol..

[10] Chen CJ, Olson M, Chen R, Lumbroso B, Huang D, Jia Y, Chen CJ, Rispoli M, Romano A, Waheed NK (2015). OCT Aangiography Examination of Type 2 Idiopathic Type 2 Telangiectasia. Clinical OCT Angiography Atlas (1st ed).

[11] Thorell MR, Zhang Q, Huang Y, An L, Durbin MK, Laron M, Sharma U, Stetson PF, Gregori G, Wang RK, Rosenfeld PJ (2014). Swept source OCT angiography of macular telangiectasia type 2. Ophthalmic Surg Lasers Imaging Retina..

[12] Gupta V, Gupta A, Dogra MR, Agarwal A (2005). Optical coherence tomography in group 2A idiopathic juxtafoveolar telangiectasis. Ophthalmic Surg Lasers Imaging..

[13] Surguch V, Gamulescu MA, Gabel VP (2007). Optical coherence tomography findings in idiopathic juxtafoveal retinal telangiectasis. Graefes Arch Clin Exp Ophthalmol..

[14] Bringmann A, Iandiev I, Pannicke T, Wurm A, Hollborn M, Wiedemann P, Osborne NN, Reichenbach A (2009). Cellular signaling and factors involved in Müller cell gliosis:neuroprotective and detrimental effects. Prog Retin Eye Res..

[15] Unterlauft JD, Eichler W, Kuhne K, Yang XM, Yafai Y, Wiedemann P, Reichenbach A, Claudepierre T (2012). Pigment epithelium-derived factor released by Müller glial cells exerts neuroprotective effects on retinal ganglion cells. Neurochem Res.

[16] Charbel Issa P, Heeren TF, Kupitz EH, Holz FG, Berendschot TT (2016). Very Early Disease Manifestations Of Macular Telangiectasia Type 2. Retina..

[17] Spaide RF, Klancnik JM, Cooney MJ, Yannuzzi LA, Balaratnasingam C, Dansingani KK, Suzuki M (2015). Volume Rendering Optical Coherence Tomography Angiography of Macular Telangiectasia Type 2. Ophthalmology..

[18] Spaide RF, Klancnik JM, Cooney MJ (2015). Retinal vascular layers in macular telangiectasia type 2 imaged by optical coherence tomographic angiography. JAMA Ophthalmol..

[19] Davidorf FH, Pressman MD, Chambers RB (2004). Juxtafoveal telangiectasis-a name change?. Retina.

[20] Soheilian M, Tavallali A, Peyman GA (2007). Identiﬁcation of intraretinal neovascularization by high-speed indocyanine green angiography in idiopathic perifoveal telangiectasia. Ophthalmic Surg Lasers Imaging..

[21] Zhang Q, Wang Rk, Chen Cl, Legarreta Ad, Durbin MK, An L, Sharma U, Stetson PF, Legarreta JE, Roisman L, Gregori G, Rosenfeld PJ (2015). Swept Source Optıcal Coherence Tomography Angiography Of Neovascular Macular Telangiectasia Type 2. Retina..

[22] Shen W, Fruttiger M, Zhu L, Chung SH, Barnett NL, Kirk JK, Lee S, Coorey NJ, Killingsworth M, Sherman LS, Gillies MC (2012). Conditional Müllercell ablation causes independent neuronal and vascular pathologies in an oveltransgenic model. J Neurosci..

